# Impact of vascular risk factor in neurodegenerative dementia: a retrospective study

**DOI:** 10.1002/alz70857_105449

**Published:** 2025-12-25

**Authors:** Sarath Govindaraj, Shivam Mishra, Suvarna Alladi, Faheem Arshad, Jitender Saini

**Affiliations:** ^1^ National Institute of Mental Health and Neurosciences, Bangalore, Karnataka, India; ^2^ National Institute of Mental Health and Neuro Sciences, Bengaluru, Karnataka, India; ^3^ National Institute of Mental Health and Neurosciences, Bengaluru, Karnataka, India

## Abstract

**Background:**

The prevalence of dementia is dramatically increasing worldwide. In this context, early diagnosis and identification of presymptomatic individuals at higher risk of developing dementia is crucial. Early interventions through modifiable risk factors can help in reducing the burden of dementia. Therefore, we aim to study the influence of vascular risk factors in the clinical profile and imaging of degenerative dementias and vascular dementia in a cohort in South India.

**Method:**

300 patients with different dementia subtypes were included from the Cognitive Disorder Clinic (CDC) registry in a tertiary care hospital in India. The presence of vascular risk factors (Hypertension, diabetes, dyslipidemia, smoking, alcohol and cardiovascular diseases), demographic and clinical profile (age of onset, Addenbrookes Cognitive Examination III (ACE III), Clinical Dementia Rating (CDR) Scale, imaging profile (visual rating scale for regional and global atrophy, white matter hyperintensities (Fazekas grading), microbleeds) were recorded and analyzed.

**Result:**

Of 300 patients evaluated, Alzheimer's Disease (AD) (132, 44%) was the most common subtype, followed by Vascular Dementia (VaD) (70, 23.3%), Dementia with Lewy Bodies (DLBD) (31, 10.3%), Mixed dementia (29, 9.7%) and other dementias [67, 22%]. Prevalence of hypertension was highest in mixed dementia (72.4%), followed by vascular dementia (70%). Diabetes (47.1%), smoking (42.9%) and alcoholism (12.9% (*p* <0.001) were higher in Vascular dementia compared to other dementias. Dyslipidemia was observed more frequently in Frontotemporal dementia (FTD) (40.7%) followed by AD (31.5%). Brain imaging demonstrated Grade 2 and Grade 3 white matter hyperintensities in VaD (12, 50%) and in FTD (67.3%) followed by AD (50%). Similarly, microbleeds were noted in vascular dementia (24%) and also to a lesser extent in mixed dementia and AD.

**Conclusion:**

These findings highlight the association between vascular risk factors in neurodegenerative dementia and vascular dementia, indicating the importance of early risk factor identification and targeted interventions. Future research should focus on longitudinal studies to better understand the dynamic interactions of these mechanisms and refine strategies for prevention and treatment across diverse dementia subtypes.

